# Longevity is associated with relative brain size in birds

**DOI:** 10.1002/ece3.2961

**Published:** 2017-04-09

**Authors:** Piotr Minias, Patrycja Podlaszczuk

**Affiliations:** ^1^ Department of Biodiversity Studies and BioeducationFaculty of Biology and Environmental ProtectionUniversity of ŁódźŁódźPoland

**Keywords:** birds, brain evolution, comparative analysis, life history, lifespan

## Abstract

Brain size of vertebrates has long been recognized to evolve in close association with basic life‐history traits, including lifespan. According to the cognitive buffer hypothesis, large brains facilitate the construction of behavioral responses against novel socioecological challenges through general cognitive processes, which should reduce mortality and increase lifespan. While the occurrence of brain size–lifespan correlation has been well documented in mammals, much less evidence exists for a robust link between brain size and longevity in birds. The aim of this study was to use phylogenetically controlled comparative approach to test for the relationship between brain size and longevity among 384 avian species from 23 orders. We used maximum lifespan and maximum reproductive lifespan as the measures of longevity and accounted for a set of possible confounding effects, such as allometry, sampling effort, geographic patterns, and life‐history components (clutch size, incubation length, and mode of development). We found that both measures of longevity positively correlated with relative (residual) brain size. We also showed that major diversification of brain size preceded diversification of longevity in avian evolution. In contrast to previous findings, the effect of brain size on longevity was consistent across lineages with different development patterns, although the relatively low strength of this correlation could likely be attributed to the ubiquity of allomaternal care associated with the altricial mode of development. Our study indicates that the positive relationship between brain size and longevity in birds may be more general than previously thought.

## 1 Introduction

Brains of higher vertebrates show immense variation in size, both in absolute terms and after controlling for the effects of allometry, and understanding the ultimate causes of this variation has become a challenging task for evolutionary biologists (Allman, 2000). It has long been recognized that the evolution of large brains is associated with both costs and benefits, and it is their net benefit which should be favored by selection (Isler & van Schaik, 2009a). Large brains are metabolically expensive, as brain tissue requires nearly an order of magnitude more energy per unit weight than many other somatic tissues (Mink, Blumenschine, & Adams, 1981). Also, in contrast to many other organs, the energetic needs of the brain cannot be temporarily reduced (Karasov, Pinshow, Starck, & Afik, 2004). As a result, metabolic costs of large brain size have to be met by either increasing the total energy budget or by compensating changes in energy allocation to other maintenance functions (so‐called expensive brain hypothesis; Isler & van Schaik, 2009a). This compensation may proceed via reductions in the costs of growth or production, forcing animals to prolong development and reproductive periods, which may in turn select for longer lifespan.

Increased longevity may also be facilitated by adaptive advantages of large brains. The hypothesis of “cognitive buffer” assumes that brains disproportionally large for a given body size are associated with elevated cognitive capacities, which help to construct behavioral responses to novel socioecological challenges (González‐Lagos, Sol, & Reader, 2010; Sol, 2009). Large brains are able to store more information on the resources in the environment, allowing to increase foraging efficiency in varying environmental conditions or to subsist on ubiquitous food during periods of environmental stress (Allman, McLaughin, & Hakeem, 1993). Large‐brained species also show higher behavioral flexibility and are more capable of social and reverse learning (Dunbar & Shultz, 2007; Lefebvre, Whittle, Lascaris, & Finkelstein, 1997). All these cognitive advantages are likely to reduce mortality and, consequently, increase lifespan (Sol, Székely, Liker, & Lefebvre, 2007).

Organismal aging is a very complex process that embraces a wide array of physiological mechanisms. Currently, production of reactive oxygen species (ROS) and cell membrane unsaturation level forms the basis of the biological hypotheses for the evolution of aging and lifespan. Both antioxidant defenses and the ability to repair oxidative damage tend to decline with old age, contributing to cellular and whole organism senescence (Metcalfe & Alonso‐Álvarez, 2010). There is evidence, also from avian research, that oxidative stress may increase in old age due to an increase in the rate of ROS production, as mtDNA accumulates more damage (Alonso‐Álvarez, Pérez‐Rodriguez, Garcia, Viñuela, & Mateo, 2010; Alonso‐Álvarez et al., 2006). Further, cell membrane fatty acid composition is an important determinant of tissue susceptibility to ROS, as saturated fatty acids are much less susceptible to peroxidation that unsaturated ones (Costantini, 2008). Thus, it has been proposed that membrane fatty acid composition evolved to cope with the detrimental effects of pro‐oxidants and several studies confirmed that this trait can explain lifespan differences between species (Hulbert, Faulks, & Buffenstein, 2006; Pamplona, Barja, & Portero‐Otin, 2002). For example, a comparison between two avian orders showed that the 3.8‐fold greater predicted longevity of Procellariformes versus Galliformes was associated with elevated contents of monounsaturated and reduced contents of polyunsaturated fatty acids, resulting in reduced peroxidation index in heart membrane lipids (Buttemer, Battam, & Hulbert, 2008). While there is no general consensus on the relative importance of different physiological mechanisms in the process of aging (Costantini, 2008), it has been acknowledged that brain participates in the stabilization of life processes of the organism and may counteract organismal aging by means of its regulatory function in the maintenance of physiological and hormonal processes (Hofman, 1983). Provided that these regulatory, as well as cognitive, benefits of large brain size outbalance metabolic costs of its development, a positive correlation between brain size and longevity would be expected.

So far, positive relationships between brain size and lifespan have been reported almost exclusively for different taxonomic groups of mammals (e.g., Allman et al., 1993; Eisenberg & Wilson, 1981; Hofman, 1993). These reports have been supported with an extensive comparative analysis of brain size in nearly half a thousand mammal species, showing that mammals with larger brains than expected for their body size tended to live longer than those with smaller brains (González‐Lagos et al., 2010). Surprisingly, much less evidence exists for a robust link between brain size and longevity in birds, although the mode of brain evolution is thought to differ between these groups of vertebrates (Isler & van Schaik, 2009b; Nealen & Ricklefs, 2001). A comparative study by Isler and van Schaik (2009b) indicated that reproductive lifespan of birds positively correlated with relative brain size in precocial, but not in altricial birds. A recent study by Sol, Sayol, Ducatez, and Lefebvre (2016) provided more convincing evidence for cognitive buffer hypothesis in birds, showing that residual brain size is directly and indirectly (via its effect on innovation propensity) associated with maximum lifespan. Nevertheless, both these studies did not control for important confounding variables, which could possibly obscure the results. The aim of this study was to examine the robustness of the association between brain size and lifespan in birds, while controlling for a set of possible confounding effects, such as allometry, sampling effort, life‐history traits, and geographic patterns. For this purpose, we applied phylogenetic comparative methods to a dataset of 384 avian species from 23 orders.

## 2 Material and Methods

### 2.1 Brain size

Following argumentation of Lendvai, Bókony, Angelier, Chastel, and Sol (2013), we have decided to use the size of the whole brain in our comparative analysis for two main reasons: (1) large availability of published data on whole‐brain volume/mass, and (2) tight correlations of whole‐brain volume with neuron density and volume of brain components, for example, associative pallium, responsible for domain‐general cognition abilities such as innovativeness and learning (Herculano‐Houzel, Collins, Wong, & Kaas, 2007; Sayol, Lefebvre, & Sol, 2016; Timmermans, Lefebvre, Boire, & Basu, 2000). Consistently, it has been shown that whole brain size positively correlates with several measures of behavioral flexibility and abilities to survive in novel ecological conditions (reviewed by Lefebvre & Sol, 2008). Data on brain mass were collected from a variety of sources (Galván & Møller, 2011; Garamszegi, Møller, & Erritzøe, 2002; Lendvai et al., 2013; Sol et al., 2010), which combined two methods of brain size measurements: (1) direct measurements of actual brain mass, and (2) measurements of endocranial volume. The latter technique estimates brain volume by filling the skull with such materials as lead shots or beads, the volume of which is then measured and converted to mass by multiplying with the density of fresh brain tissue (1.036 g/ml; Iwaniuk & Nelson, 2002). Although combining brain size estimates obtained with different methods has raised concerns (Healy & Rowe, 2007), several studies reported very strong correlations between brain sizes estimated by the endocranial technique and those estimated by weight (Iwaniuk & Nelson, 2002; Overington, Morand‐Ferron, Boogert, & Lefebvre, 2009; Sol et al., 2010). It has also been shown that brain size estimates are highly repeatable between different published sources (Garamszegi et al., 2002) and that combination of estimates obtained with different techniques in one analysis does not alter the conclusions (e.g., Overington et al., 2009). Brain mass was averaged over sexes prior to analyses, as the sizes of male and female brains are known to tightly coevolve (Garamszegi, Eens, Erritzøe, & Møller, 2005).

It has been long recognized that adaptation for enhanced neural processing is better indicated by brain size corrected for body size rather than by brain size per se (Jerison, 1973). There are a few alternative methods used to remove the allometric effect of body size on brain size (Deaner, Nunn, & van Schaik, 2000), but some of them (e.g., calculating the fraction of body mass that corresponds to brain mass) have been reported not to remove the effect of body size appropriately. Thus, following recommendations by Sol et al. (2007), we extracted residuals of brain size against body mass using log–log least‐square linear regression (*R* ^2^ = .92, *p *<* *.001), which remains among the most popular methods of removing allometry in brain size (Galván & Møller, 2011; Lendvai et al., 2013; Sol, Lefebvre, & Rodríguez‐Teijeiro, 2005; Sol & Price, 2008). Following Sol et al. (2016), we also calculated brain size residuals with phylogenetic corrected least‐square regression using the Analysis of Phylogenetics and Evolution (APE) package (Paradis, Claude, & Strimmer, 2004) developed for R statistical environment (R Development Core Team, 2013). Residuals calculated using phylogenetic and nonphylogenetic methods produced virtually identical results of comparative analyses, and thus, only results based on nonphylogenetic brain size residuals (henceforth referred to as “residual brain size”) are reported. Data on body masses were compiled from standard resources (Cramp & Perrins, 1977‐1994; del Hoyo, Elliott, & Sargatal, 1992‐2011), as well as from other comparative studies on birds (e.g., Galván & Møller, 2011; Garamszegi et al., 2002). Male and female body masses were averaged for size‐dimorphic species prior to analysis.

### 2.2 Lifespan and reproductive lifespan

We used maximum lifespan as a basic measure of longevity, as it is thought to best reflect the aging rate in vertebrates (de Magalhães, Costa, & Church, 2007). Data on maximum lifespan were collected using the AnAge database (de Magalhães & Costa, 2009), which features nearly 1,200 avian species and has been commonly used as a central resource for comparative analyses of vertebrate longevity (e.g., Capellini, Baker, Allen, Street, & Venditti, 2015; Cooper, Kamilar, & Nunn, 2012; Healy et al., 2014). The database contains the highest reported values of maximum lifespan, but excludes records based on single or a few individuals (de Magalhães & Costa, 2009). Data on maximum lifespan were also used to calculate reproductive lifespan (duration of reproductive life) for each species. For this purpose, we collected data on the age at first reproduction for each species and subtracted it from longevity records, following recommendations of Barrickman, Bastian, Isler, and van Schaik (2008).

Longevity records may provide reliable information on lifespan only if adjusted for sampling effort, as probability of recording an extremely old individual increases with sample size (Møller, 2007). To control for the sampling effort, we extracted information on sample size from the AnAge database, where it was categorized into three groups (small, medium, and large). Also, we recorded whether maximum longevity was measured in captivity or in the wild, as data obtained from these two sources may not be equivalent. For example, it has been shown that maximum lifespan recorded for nearly 500 species of mammals was significantly longer in captive animals, when compared to wild individuals (González‐Lagos et al., 2010). Thus, the source of data was included as a fixed factor in all the models.

Variation in body mass is one of the major causes of bias in the comparative analyses of senescence, as large species generally have greater longevity, survive better, and start their first reproduction at an older age than smaller species (Møller, 2007). Thus, similarly to brain size, lifespan needs to be adjusted for allometry in comparative analyses. However, using the same dataset of body mass estimates to allometrically scale brain size and lifespan may increase the chance of type *I* errors, as residuals of the response and predictor variables can be biased in the same direction if compiled body masses under‐ or overestimate the true values (Barrickman et al., 2008; González‐Lagos et al., 2010). For this reason, we compiled a second independent set of body masses using the AnAge database. As body mass explained only moderate proportion of variance in the two measures of lifespan (*R* ^2^ = .53, *p *<* *.001 for lifespan; *R* ^2^ = .49, *p *<* *.001 for reproductive lifespan), we entered log‐transformed lifespan measures as the dependent variables in the comparative models, while the log‐transformed body mass was entered as an independent variable to control for allometry. Residuals of log–log least‐square regressions of lifespan and reproductive lifespan against body mass (henceforth referred to as “residual lifespan” and “residual reproductive lifespan,” respectively) were used in the analysis of phylogenetic autocorrelation.

### 2.3 Confounding variables

Evolutionary relationship between lifespan and other life‐history traits is thought to be primarily mediated by variation in body size. In general, large‐bodied species live slower lives, which means that they not only live longer, but also become sexually mature at older age, have longer egg incubation or gestation periods, and produce smaller clutches or litters (Promislow & Harvey, 1990). However, it has been shown that some life‐history traits still correlate across taxa even when the effect of body size is held constant (Harvey & Clutton‐Brock, 1985; Read & Harvey, 1989). It has been proposed that different species may lie at different points along the fast–slow continuum, reflecting the extent to which species are subject to density‐independent versus density‐dependent selection (Promislow & Harvey, 1990). While density‐dependent selection is thought to act stronger on large species, both types of selection may also operate independently of size (Ross, 1988). Other studies indicated that after removing the body size effects, the speed of life may vary along two major axes reflecting: (1) how species balance offspring size against offspring number, and (2) the timing of reproductive bouts (Bielby et al., 2007). Consequently, in our comparative analysis, we controlled for incubation length and clutch size, two basic life‐history components associated with the speed of life in birds (Martin, 2002). Data on incubation length and clutch size were compiled from the AnAge database and standard resources (Cramp & Perrins, 1977‐1994; del Hoyo et al., 1992‐2011).

Longevity may also show specific geographic patterns. First, slow life histories are likely to predominate at low latitudes, as animals in the tropics are generally known to invest more in self‐maintenance, hence extending reproductive life (Murphy, 1968). Second, longevity of species from high latitudes may be reduced by the higher rate of density‐independent mortality due to abiotic factors (Forsman & Mönkkönen, 2003; Newton, 1998). In fact, a comparative analysis of birds showed weak, but significant relationship between senescence and latitude (Møller, 2007) and, thus, we have decided to control for the effect of latitude in our analyses. For this purpose, we extracted the northernmost and the southernmost distribution limits of the breeding range to the nearest 0.1° (BirdLife International, 2016; Cramp & Perrins, 1977‐1994). Mean breeding latitude was expressed as an average from the two distribution limits.

**Figure 1:**
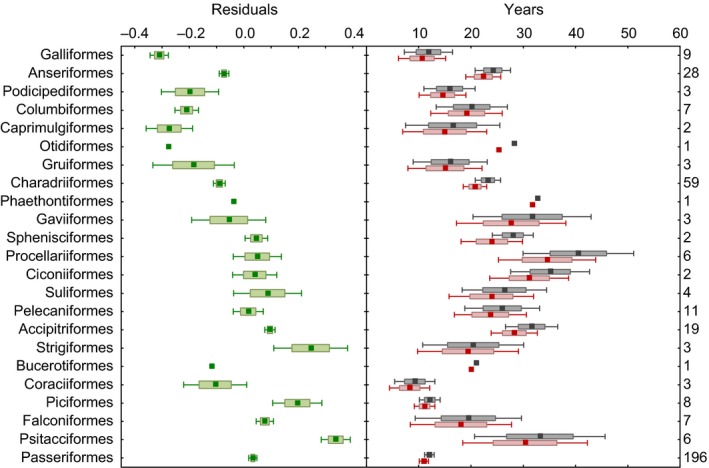
Residual brain size (green), lifespan (gray), and reproductive lifespan (red) in 23 avian orders. Point—mean, box—*SE*, whiskers—1.96*SE. Sample sizes are shown at the right vertical axis

As predicted by the “expensive brain” hypothesis, the costs of large brain size must be met by an increase in the total energy turnover or reduction in energy allocation to other basic functions, such as maintenance or production (Isler & van Schaik, 2009a). Consistently, it has been suggested that allomaternal energy inputs during offspring production might be crucial for the evolution of large brain size in birds and mammals (Isler & van Schaik, 2009b). This prediction was supported by the trade‐off between the maximum rate of population increase (*r*_max_) and brain size that was found exclusively in precocial species, but not in altricial species where the mother's energetic burden during reproduction is alleviated by helpers (Isler & van Schaik, 2009b). To control for the variation in the allomaternal energy inputs during reproduction, we collected data on the mode of chick development, which was categorized into four groups: precocial, semi‐precocial, semi‐altricial, and altricial. Full information on longevity, brain size, and all confounding variables were collected for 384 bird species from 23 orders (Figure 1).

### 2.4 Comparative analysis

Data for different species in a comparative analysis are statistically dependent, as closely related species are more likely to have similar phenotypes because of the shared ancestry. In order to control for the shared evolutionary history, we conducted phylogenetic generalized least squares (PGLS) regression, which incorporates a matrix of the expected covariances among the species based on their phylogenetic relationships (Martins & Hansen, 1997). This approach allowed us to estimate the importance of phylogenetic correlations by calculating the phylogenetic scaling parameter λ, which is used as the branch‐length transformation in the regression analysis (Pagel, 1999). In general, λ varies between zero and one, where zero indicates phylogenetic independence (all branches collapse to zero), and a value of one indicates that trait evolution corresponds to a Brownian motion model (the internal branch length of the phylogeny remains untransformed) (Freckleton, Harvey, & Pagel, 2002). Brownian motion is a good approximation of evolution by purely random genetic drift, but may not be appropriate when there is consistent selection toward a single optimum trait value, which is better modeled by an Ornstein–Uhlenbeck (OU) process (Martins, 1994). For our measures of lifespan, the Brownian motion model (adjusted for λ) performed better than the OU model (relative fit of models measured with the difference in the Akaike's Information Criterion; ΔAIC = 14.4 for lifespan; ΔAIC = 10.6 for reproductive lifespan). Thus, the Brownian motion model was used in all further comparative analyses, and the phylogenetic scaling parameter λ was set to its maximum likelihood estimate that had been evaluated separately for each model.

Phylogenetic relationships between species were reconstructed using the most recent complete avian time‐calibrated phylogeny (Jetz, Thomas, Joy, Hartmann, & Mooers, 2012) with a backbone tree developed by Ericson et al. (2006). Pseudoposterior distributions of phylogenetic trees were downloaded from the BirdTree databse (http://www.birdtree.org; Jetz et al., 2012), which has become the standard approach for accommodating uncertainty in downstream comparative analyses (Rubolini, Liker, Garamszegi, Møller, & Saino, 2015). One thousand alternative trees were summarized into a single consensus tree using the SumTrees program that is a part of DendroPy, a Python library for phylogenetic computing (Sukumaran & Holder, 2010). To obtain a consensus tree, we adopted a majority rule, where a branching event is considered supported if it occurs in >50% of the input trees (Holder, Sukumaran, & Lewis, 2008). The mean branch lengths of the consensus tree were adjusted such that the ages of the subtended nodes corresponded to the median age of the corresponding nodes of the input trees.

All PGLS analyses were conducted using the APE package. Lifespan and reproductive lifespan were included as the dependent variables in the separate PGLS models. Residual brain size, log body mass, clutch size, incubation length, and latitude were included as covariates, while mode of development, sampling effort, and data source was entered as fixed factors. We also included an interaction between residual brain size and mode of development, to test whether the effect of brain size on longevity is consistent across lineages with different development patterns. To obtain more parsimonious reduced models, we removed nonsignificant (*p* > .10) predictors from the initial full models. Effect sizes were estimated with the partial eta squared calculated for nonphylogenetic reduced models using the glm2 (Marschner, 2011) and lsr (Navarro, 2015) packages. The role of evolutionary history in explaining the current‐day variation in lifespan and brain size was evaluated with two different methods, following Sol et al. (2007). First, we used nested ANOVA to identify the taxonomic level associated with major diversification in these traits, which was performed in Statistica 10.0 (StatSoft, Tulsa, OK, USA). Second, we used the spatial autocorrelation statistic Moran's *I* (Gittleman & Kot, 1990) to assess phylogenetic autocorrelation in residual lifespan, residual reproductive lifespan, and residual brain size. Phylogenetic correlograms of normalized Moran's *I* (*I*/*I*_max_) were used to assess the strength of autocorrelation in the traits at different taxonomic levels (genus, family, and order). The analysis of Moran's *I* was based on the phylogenetic classification proposed by Winkler, Billerman, and Lovette (2015) and conducted using the APE package. All values are reported as means ± *SE*.

## 3 Results

**Figure 2:**
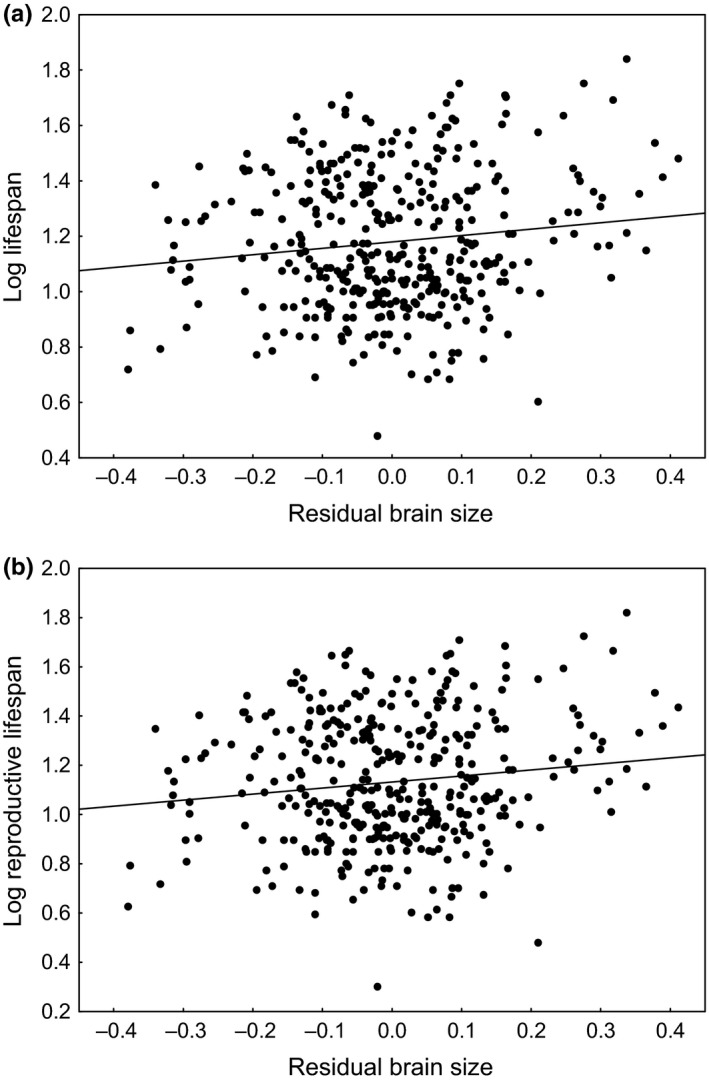
Relationship of residual brain size with lifespan (a) and reproductive lifespan (b) among 384 bird species from 23 orders

Our comparative analysis identified residual brain size as a significant predictor of longevity in birds. Both the reduced models (Table 1) showed that residual brain size was positively related to lifespan (β = 0.207 ± 0.086; Figure 2a) and reproductive lifespan (β = 0.237 ± 0.094; Figure 2b). The effect sizes of residual brain size were relatively low (0.044 for lifespan, 0.040 for reproductive lifespan). In these analyses, we controlled for body mass, basic life‐history traits, latitude, sampling effort, as well as we accounted for the source of information (captive vs. wild populations) on longevity records. We found no evidence for the effect of incubation, clutch size, mode of development, and latitude on longevity in birds (Table 1). Also, we found that relationships of residual brain size and the two measures of longevity were consistent across lineages with different modes of development, as indicated by nonsignificance of appropriate interactions (Table 1). All the models gave an indication for the positive impact of sampling effort on longevity records (large vs. small sample size: β = 0.120 ± 0.032 for lifespan; β = 0.132 ± 0.036 for reproductive lifespan). Longevity records were also significantly higher for captive individuals when compared with wild populations (β = 0.117 ± 0.028 for lifespan; β = 0.116 ± 0.031 for reproductive lifespan). The phylogenetic signal in the data was moderate, as indicated by λ = 0.33 for lifespan and λ = 0.28 for reproductive lifespan.

**Table 1:** Full and reduced models for lifespan and reproductive lifespan in birds. Significant predictors are marked in bold

Predictor	Lifespan		Reproductive lifespan	
	*F*	*p*	*F*	*p*
Full model				
Intercept	**74.77**	**<.001**	**60.31**	**<.001**
Residual brain size (RBS)	3.15	.077	3.17	.076
Mode of development (MoD)	2.53	.057	2.28	.079
RBS*MoD	0.97	.41	0.94	.42
Incubation	0.40	.53	0.02	.88
Clutch size	0.94	.33	0.97	.33
Latitude	0.11	.74	0.02	.89
Body mass	**55.13**	**<.001**	**49.49**	**<.001**
Data source	**18.68**	**<.001**	**14.89**	**<.001**
Sampling effort	**7.58**	**<.001**	**7.40**	**<.001**
Reduced model				
Intercept	**109.9**	**<.001**	**87.76**	**<.001**
Residual brain size	**5.80**	**.017**	**6.36**	**.012**
Mode of development	2.49	.060	1.83	.14
Body mass	**77.25**	**<.001**	**66.26**	**<.001**
Data source	**17.29**	**<.001**	**13.79**	**<.001**
Sampling effort	**8.00**	**<.001**	**7.83**	**<.001**

**Figure 3:**
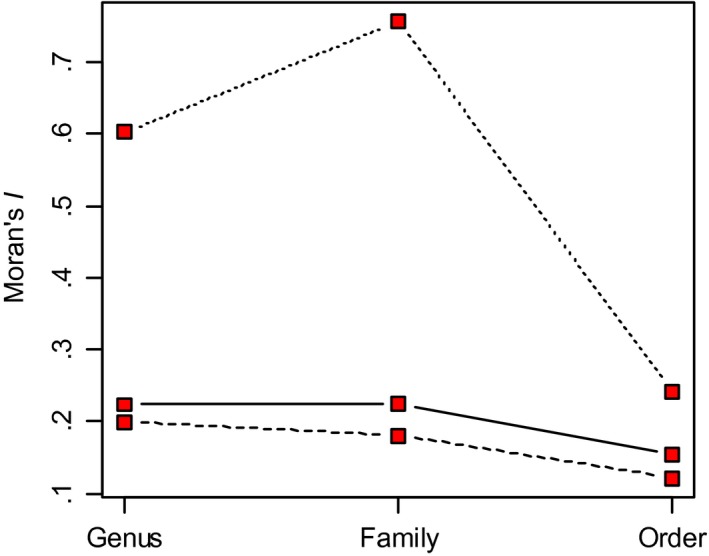
Phylogenetic correlogram for residual brain size (dotted line), residual lifespan (solid line), and residual reproductive lifespan (dashed line). Moran's *I* is shown for three taxonomic levels (genus, family, order)

We found that higher taxonomic levels (family and order) explained relatively small proportion of variance in residual lifespan (15.9% and 25.4%, respectively) and residual reproductive lifespan (16.2% and 21.2%, respectively), suggesting that most diversification in these traits occurred relatively late in avian radiation. Family and order explained much larger proportion of variance in residual brain size (27.5% and 56.4%, respectively). Consistently, there was a positive phylogenetic autocorrelation at the species level in residual brain size among birds (*p* = .007), whereas no phylogenetic correlation was recorded in residual lifespan (*p* = .66) and residual reproductive lifespan (*p* = .56). Strong phylogenetic correlation in residual brain size was also recorded at the level of genus and family, while it was considerably lower at the level of order (Figure 3). In contrast, there was weak phylogenetic correlation at all taxonomic levels in residual lifespan and residual reproductive lifespan (Figure 3).

## 4 Discussion

Our comparative analyses gave support for a robust positive relationship between relative brain size and longevity in birds. These results complement the previous findings of Sol et al. (2007), who showed that avian species with larger brains relative to their body size have higher survival rate in nature. Taking into account that similar correlations between brain size and longevity have been reported for mammals (González‐Lagos et al., 2010), we may conclude that this relationship is likely to be a general pattern among higher vertebrates.

There are several nonexclusive mechanisms which can explain a positive evolutionary relationship between lifespan and brain size. While current evidence is insufficient to determine which of these mechanisms provides a primary explanation for the evolution of large brains (reviewed in Sol, 2009), the cognitive buffer hypothesis has received the most solid empirical support. The basic assumption of the cognitive buffer hypothesis is that the primary adaptive function of a large brain is to facilitate the construction of behavioral responses against novel socioecological challenges through general cognitive processes such as innovation or learning (Sol, 2009). This behavioral flexibility is expected to reduce mortality, especially during the periods of environmental stress, and consequently increase lifespan. Indeed, there is increasing empirical evidence that brain size, as well as the relative size of association areas in the brain, positively correlate with the frequency of novel behaviors observed in nature (Lefebvre, Reader, & Sol, 2004; Lefebvre et al., 1997) and with innovation diversity (Overington et al., 2009). It has also been shown that large‐brained birds survive better when introduced to a novel environment, where individuals are likely to experience many novel challenges and their survival should strictly depend on whether they are able to rapidly develop novel behavioral responses (Sol, Duncan, Blackburn, Cassey, & Lefebvre, 2005). Other studies showed that large‐brained birds are generally more tolerant to habitat alterations (Schultz, Bradbury, Evans, Gregory, & Blackburn, 2005) and climatic variability (Schuck‐Paim, Alonso, & Ottoni, 2008).

While not ruling out cognitive buffer effect, recent study on mammals provided convincing evidence for an alternative hypothesis on the evolutionary association between brain size and longevity (Barton & Capellini, 2010). It has been shown that evolutionary changes in pre‐ and postnatal brain growth correlated specifically with duration of the relevant phases of maternal investment (gestation and lactation, respectively). Also, after accounting for the duration of maternal investment, adult brain size was uncorrelated with other life history traits such as lifespan (Barton & Capellini, 2010). Consequently, it has been concluded that the general pattern of slow life histories in large‐brained mammal species could be a direct consequence of developmental costs, being consistent with the “expensive brain” hypothesis (Isler & van Schaik, 2009a).

Although the hypotheses of cognitive buffer and expensive brain assume that the evolution of larger brains drives the evolution of longevity, it has to be kept in mind that the brain size–lifespan relationships are of correlative nature, meaning that the reverse causality cannot be ruled out. In fact, slow life histories characterized by delayed maturation and longer lifespan give parents the opportunity of a prolonged investment in offspring (Covas & Griesser, 2007) and increase the time available for brain growth (Kaplan, Hill, Lancaster, & Hurtado, 2000; Walker, Burger, Wagner, & Von Rueden, 2006). Also, longer lifespan increases probability that an individual encounters severe crises during its lifetime and, thus, longevity should favor selection for enlarged brains in order to sustain animals through the periods of environmental stress or any other life‐threatening situations (Allman et al., 1993). A framework that integrates these scenarios suggests that brain size may both affect and be affected by life‐history strategies, implying a positive feedback in the evolution of large brains and longevity (Sol et al., 2007). While this hypothesis remains to be tested, our results confirm previous findings that most diversification in residual brain size occurred relatively early in avian radiation (Isler & van Schaik, 2009b; Sol et al., 2007), as indicated by strong phylogenetic signal and spatial autocorrelation (estimated with Moran's *I*) in this trait. By contrast, phylogenetic signal in residual lifespan and residual reproductive lifespan was weak and phylogenetic autocorrelation was low at all taxonomic levels. This indicates that major diversification in brain size preceded diversification in longevity and, thus, cannot be perceived as its mere evolutionary consequence. Similar conclusions have been reached by Sol et al. (2007), who compared diversification patterns of brain size and mortality rates in birds. Also, an analysis of causal scenarios with phylogenetic path analysis indicated that lifespan of birds is directly affected by innovation propensity, while both are associated by the indirect and common effects of relative brain size (Sol et al., 2016).

In contrast to mammals, avian residual brain size explained only a small proportion (ca. 4%) of variance in lifespan and reproductive lifespan. Extensive comparative analysis of 18 mammal orders showed that 13% of variance in residual lifespan was explained by residual brain size, while controlling for phylogeny. Effect sizes for this relationship in some groups of mammals have been reported to be much higher, for example, in primates brain size explained over 40% of variance in longevity (Allman et al., 1993). As suggested by Isler and van Schaik (2009b), this apparent discrepancy between birds and mammals can be associated with general differences in developmental patterns among these two vertebrate classes. Ca. 90% of all bird species show biparental or cooperative care, which is associated with altricial mode of development and substantial allomaternal help during the period of offspring production (Cockburn, 2006). By contrast, allomaternal care is relatively uncommon in mammals and has evolved in just a few taxonomic groups, such as rodents, carnivores, and primates (Kleiman, 1977). According to the expensive brain hypothesis (Isler & van Schaik, 2006), there should be an evolutionary trade‐off between an investment in large brain and investment in growth or reproduction, resulting in relatively slow development, increased lifespan, and reduced fertility of animals with brains larger than expected for their size. Nevertheless, the life‐history compensation for energetic costs of producing and maintaining large brains is expected to weaken or disappear when mothers receive help from conspecifics during offspring production (Isler & van Schaik, 2009b), as the allomaternal energy input allows for increased fertility and faster offspring development. Consistently with this prediction, Isler and van Schaik (2009b) found no correlation between relative brain mass and maximum lifespan or fertility in altricial birds, while both these life‐history traits strongly correlated with relative brain mass in precocial birds. In this study, we failed to corroborate the effect of the mode of development on the brain size–lifespan relationship, which could be due to several methodological differences in our analytical approach, such as (1) removing body size effects separately for brain size and lifespan estimates; (2) using the PGLS approach, which is known to much better accommodate phylogenetic signal to the data when compared with independent contrasts; (3) controlling lifespan estimates for several confounding variables. While our results suggest that the positive relationship between brain size and longevity in birds may be more general than previously thought, we acknowledge that the relatively low strength of this correlation is likely to result from the ubiquity of allomaternal care associated with altricial mode of development.

## Conflict of Interest

None declared.

## Data Accessibility

The raw data have been supplied as an Table S1.
